# Early sacubitril/valsartan use associated with reduced atrial fibrillation risk in patients with acute myocardial infarction complicated by mitral regurgitation: a retrospective cohort study

**DOI:** 10.3389/fcvm.2025.1658746

**Published:** 2025-09-26

**Authors:** Hongtao Yin, Yanqing Zhou, Yingjun Zhou, Lin Ren, Lixiang Ma

**Affiliations:** ^1^ Department of Cardiology, First Hospital of Qinhuangdao, Qinhuangdao, China; ^2^ Department of Operating Room, First Hospital of Qinhuangdao, Qinhuangdao, China

**Keywords:** acute myocardial infarction, mitral regurgitation, sacubitril/valsartan, atrial fibrillation, propensity score matching

## Abstract

**Background:**

Patients with acute myocardial infarction (AMI) complicated by mitral regurgitation (MR) experience a substantially elevated risk of atrial fibrillation (AF). Evidence remains limited regarding whether early administration of sacubitril/valsartan confers additional protection against new-onset AF in this high-risk population to traditional ACEI/ARB therapy.

**Objective:**

To evaluate the impact of early sacubitril/valsartan therapy on the 1-year cumulative incidence of AF in patients with AMI complicated by MR.

**Methods:**

In this single-center retrospective cohort, 1,065 in-patients with AMI complicated by MR (June 2021–December 2023) were categorized by discharge prescription into the Sacubitril/Valsartan group (*n* = 427) or the ACEI/ARB group (*n* = 638).The primary endpoint was new-onset AF within 1 year. Cumulative incidence was estimated using the cumulative incidence function (CIF) and compared with Gray's test. Associations were evaluated using Fine–Gray subdistribution hazard models [subdistribution hazard ratios (sHR)] and multivariable Cox proportional hazards models [hazard ratios (HR)]. To mitigate confounding, 1:1 propensity score matching (PSM) was performed, and analyses were repeated in the matched cohort. Prespecified subgroup analyses were performed to assess treatment-effect consistency.

**Results:**

Despite a higher baseline risk profile in the Sacubitril/Valsartan group, the 1-year cumulative incidence of AF was lower than in the ACEI/ARB group (CIF 10.8% vs. 17.9%; Gray's test *P* = 0.002). In competing-risk analysis, sacubitril/valsartan was associated with a reduced risk of AF (sHR = 0.56; 95% CI 0.39–0.81; *P* = 0.002), concordant with the multivariable Cox model (HR = 0.55; 95% CI 0.38–0.81; *P* = 0.003). After PSM, the association persisted in both the competing-risk analysis (sHR = 0.63; 95% CI 0.40–0.94; *P* = 0.025) and the Cox model (HR = 0.63; 95% CI 0.41–0.96; *P* = 0.030). Subgroup analyses demonstrated consistent benefits across all prespecified strata.

**Conclusion:**

Early sacubitril/valsartan use was associated with reduced 1-year AF risk vs. ACEI/ARB in AMI complicated by MR, suggesting a potential role in this population; prospective trials are needed to confirm causality.

## Introduction

Mitral regurgitation (MR) is one of the most common complications of acute myocardial infarction (AMI), with an incidence of 20% to 50% (1). This complication not only significantly affects short-term prognosis but is also closely associated with adverse long-term outcomes (2), increasing the risk of atrial fibrillation (AF) by 2 to 3 times (3, 4). The pathogenic mechanisms involve multiple pathophysiological features: First, MR leads to abnormal left ventricular filling patterns, which result in sustained elevations in left atrial pressure (5), providing an electrophysiological substrate for the development of AF. Second, the inflammatory response activated following myocardial infarction may promote AF by upregulating the expression of Toll-like receptors 2 and 4 (6). Additionally, remodeling of the ventricular-atrial coupling can further exacerbate both electrical and structural remodeling of the atria (7, 8). Therefore, enhanced monitoring and management of AF (4) are warranted in patients with AMI, particularly those with concomitant MR, to improve clinical outcomes.

The PARADISE-MI study (9–13) provided important evidence for evaluating sacubitril/valsartan [angiotensin receptor–neprilysin inhibitor (ARNI)] in high-risk AMI populations. In this prospective multicenter randomized trial, the primary composite endpoint—cardiovascular death, heart failure hospitalization, or outpatient symptomatic heart failure—did not differ significantly between sacubitril/valsartan and ramipril (*P* = 0.17) (14). However, a prespecified expanded secondary composite endpoint that additionally included recurrent myocardial infarction and stroke showed an approximately 16% relative risk reduction with sacubitril/valsartan (*P* < 0.05). Notably, prespecified subgroup analyses indicated that in patients aged ≥65 years and those undergoing emergency percutaneous coronary intervention, improvements in the primary endpoint reached statistical significance (10), suggesting that these higher-risk cohorts may derive more pronounced therapeutic benefit.

Moreover, a specific study focusing on patients with AMI complicated by moderate to severe MR revealed that the early initiation of sacubitril/valsartan not only effectively reduced the severity of valvular regurgitation but also decreased the risk of heart failure-related rehospitalization by 42% (15). Although current guidelines recommend the routine use of angiotensin-converting enzyme inhibitors or angiotensin receptor blockers (ACEI/ARB) in all AMI patients without contraindications (16, 17)—potentially reducing the risk of AF after AMI—there remains a lack of direct evidence regarding whether sacubitril/valsartan is superior to ACEI/ARB in improving the incidence of AF in AMI patients with concomitant MR (diagnosed during post-MI hospitalization). Therefore, targeted research is urgently needed to clarify its potential mechanisms and clinical value.

## Methods

This single-center retrospective cohort study involved patients with a AMI complicated by MR who were admitted to a specialized Cardiac Care Unit (CCU) in a tertiary teaching hospital meeting China's medical institution standards, between June 2021 and December 2023.Patients were divided into two groups based on their discharge prescriptions: the Sacubitril/Valsartan treatment group and the ACEI/ARB treatment group. Research data were collected through the hospital's electronic medical records system, outpatient follow-up records, and structured telephone follow-up utilizing standardized questionnaires. The study protocol received approval from the Institutional Ethics Committee at a tertiary teaching hospital in China (approval number: 2022C010), in strict accordance with the ethical principles outlined in the Declaration of Helsinki, and informed consent was obtained from all participants.

### Definition and grading of MR

MR is defined as the pathological backflow of blood from the left ventricle into the left atrium during systole. Following the 2020 ASE/ACC/AHA and 2021 ESC/EACTS guidelines, MR severity is determined using an integrated approach that prioritizes quantitative metrics and is corroborated by semi-quantitative and supportive evidence, avoiding reliance on any single parameter. The core quantitative metrics are regurgitant volume (RVol, ml/beat), regurgitant fraction (RF, %), and effective regurgitant orifice area (EROA, mm^2^). Semi-quantitative/supportive metrics include vena contracta (VC) width (mm), continuous-wave Doppler spectral density and triangular waveform, pulmonary venous flow pattern (with systolic flow reversal supporting at least moderate-to-severe MR), and structural remodeling such as left atrial/left ventricular enlargement, mitral annular dilation, and a prominent color jet. Grading thresholds are applied as follows: severe MR is defined by RVol ≥ 60 ml or RF ≥ 50%, typically with supportive findings such as EROA ≥ 40 mm^2^, VC ≥ 7 mm, and systolic pulmonary venous flow reversal; moderate MR is defined by RVol = 30–59 ml or RF = 30%–49%, typically supported by EROA = 20–39 mm^2^ and VC = 3.0–6.9 mm; mild MR is defined by values below these thresholds without supportive evidence of moderate or severe disease. Final grade assignment requires concordance of at least two primary metrics (usually including RVol and RF) with the supportive evidence; in cases of discordance, the overall physiologic coherence—such as left atrial enlargement and systolic pulmonary venous flow reversal—takes precedence.

### Study population

Eligible patients were those who met the study's predefined inclusion criteria for AMI. For each enrolled patient, the index transthoracic echocardiogram (Index TTE) was defined as the first high-quality TTE performed within 72 h after confirmed AMI. If only a rapid bedside assessment of insufficient quality was available within 72 h, the first TTE meeting quantitative requirements within the first week of hospitalization was designated as the Index TTE. All echocardiographic studies were retrieved from the institutional electronic medical records and picture archiving and communication system (PACS). Original images of the Index TTE were retrospectively re-evaluated by two senior echocardiographers (each with >5 years of experience) using a standardized quantitative protocol and pre-specified worksheets, blinded to clinical group allocation and outcomes (including ARNI use and incident atrial fibrillation). Discrepancies were adjudicated by a third senior reader.

Patients with AMI admitted to the cardiac care unit (CCU) were screened according to the following inclusion and exclusion criteria. The inclusion criteria were as follows: (1) fulfillment of the global universal definition of AMI (18); (2) the presence of segmental wall motion abnormalities (e.g., hypokinesia or akinesia) on echocardiography; and (3) presence of MR (mild, moderate, or severe) on echocardiography during hospitalization (19). The exclusion criteria included: (1) history of valvular heart disease, heart failure, various cardiomyopathies, or prior cardiac surgery; (2) prior diagnosis of significant MR on routine examinations; (3) patients who underwent surgical treatment for AMI complicated by mitral valve prolapse or chordae tendineae rupture; (4) presence of AF prior to admission or at discharge; (5) severe hepatic, renal, or pulmonary dysfunction, severe anemia, or advanced malignancies; (6) died during hospitalization; (7) not prescribed sacubitril/valsartan or ACEI/ARB therapy at discharge; and (8) other reasons.

### Covariates

Covariates relevant to the study were collected based on established risk factors for AF (20, 21) and the baseline clinical data of hospitalized patients. These encompassed the following dimensions: (1) demographic characteristics (age, sex, body mass index); (2) comorbidities and risk factors (hypertension, diabetes mellitus, smoking history, coronary artery disease, stroke, chronic kidney disease, and chronic obstructive pulmonary disease); (3) laboratory measurements (lipid profile, glucose metabolism, renal function, inflammatory markers, and cardiac injury biomarkers); (4) cardiac structure and function parameters (left ventricular ejection fraction, natriuretic peptide levels, heart rate, and blood pressure); (5) features related to myocardial infarction (infarction type, coronary intervention, Killip classification, and severity of MR); and (6) discharge medication regimens (antiplatelet agents, statins, beta-blockers, diuretics, and hypoglycemic agents). All covariates were systematically recorded using standardized data collection procedures and were included in multivariable adjustment models for subsequent analyses.

### Sample size

Based on historical data from our hospital's follow-up system, the 1-year incidence of AF was 6.5% among patients with AMI complicated by MR treated with oral sacubitril/valsartan, vs. 13.8% among comparable patients treated with an ACEI/ARB. Using PASS 15 and a two-proportion test with parameters set to *α* = 0.05 and *β* = 0.10 (90% confidence level, 1−*β*, and power 0.90), we estimated that a minimum of 355 per group was required. The actual sample size in each group exceeded this minimum.

### Follow-up and outcomes

Patients were stratified by the index discharge prescription into two exposure groups—ARNI vs. ACEI/ARB. The index date was uniformly defined as the day of hospital discharge for all participants. Follow-up commenced at this time-zero and continued until the earliest of incident AF, death, loss to follow-up, or administrative end at 1 year after discharge. All analyses adhered to the intention-to-treat principle, with participants retained in their initial exposure group irrespective of subsequent treatment changes or discontinuation.

Individual follow-up time was calculated in days and converted to person-years using 365 days per year; group-level person-time was the sum of individual person-years.

A standardized outpatient follow-up schedule was implemented at 1, 3, 6, and 12 months, and a routine 12-lead electrocardiogram (ECG) was performed at every visit irrespective of symptoms. ambulatory ECG (Holter) monitoring was triggered via two pathways: symptom-driven and protocol-driven. In the symptom-driven pathway, patients presenting with palpitations, dizziness, syncope, unexplained dyspnea, or worsening heart failure underwent an ECG first; if AF was not detected, a 24–48-hour Holter was subsequently arranged. In the protocol-driven pathway, to screen for silent AF, one routine 24–48-hour Holter was recommended at 6–12 months visit. Given the single-center, retrospective, real-world design, the protocol did not mandate implantable loop recorders (ILRs); however, tracings from portable or event recorders obtained during unscheduled encounters were accepted as sources for event adjudication.

AF was defined as an irregularly irregular rhythm without discernible P waves lasting ≥30 s on 12-lead ECG or Holter; atrial flutter was explicitly excluded. All putative AF events underwent blinded, independent review by two cardiologists, with discrepancies resolved by a third adjudicator. The primary outcome was the 1-year cumulative incidence of AF, encompassing paroxysmal, persistent, and permanent AF; events were confirmed by immediate ECG or ambulatory monitoring and could be ascertained during symptom-prompted assessments or incidentally through routine surveillance of asymptomatic individuals.

Safety outcomes, including hypotension, worsening renal function, hyperkalemia, and angioedema, were reported as incidence proportions with corresponding *P*-values.

### Statistical analysis

We handled missing covariate data using multiple imputation by chained equations. In this study, multivariate Cox proportional hazards regression analysis was employed to evaluate the independent association between sacubitril/valsartan and the occurrence of AF, and different models were constructed by stepwise adjustment of covariates. The cumulative incidence of AF was presented using cumulative incidence function(CIF) curves, and the Gray's test was used to compare differences between groups. Continuous variables were described as mean ± standard deviation or median (interquartile range) according to their distribution types. Chi-square test (for categorical variables), independent samples *t*-test (for normally distributed data), or Mann–Whitney *U*-test (for non-normally distributed data) were used for inter-group comparisons. To evaluate the dose–response relationship between baseline n-terminal pro-b-type natriuretic peptide (NT-proBNP) and incident AF, we fitted Cox proportional hazards models with restricted cubic splines. Overall and nonlinear components were assessed with Wald tests, and models were adjusted for the same covariates as in the primary analysis (Table 2). The proportional hazards assumption was examined using Schoenfeld residuals, with the global test indicating no violation.All statistical analyses were performed using IBM SPSS (version 26.0) and R (version 4.3.2). The threshold for statistical significance was defined as a two-sided *P* < 0.05. We assessed the proportional hazards assumption for the Cox proportional hazards model by global tests of Schoenfeld residuals. Figures were generated with R and GraphPad Prism (version 9.0).

### Sensitivity analysis

To verify the robustness of the findings, this study used propensity score matching (PSM) (22) for sensitivity analysis. Propensity scores were generated based on a multifactorial logistic regression model with matching variables covering demographic characteristics (age, gender, BMI, etc.), co-morbidities status (hypertension, diabetes mellitus, etc.), cardiac function parameters [left ventricular ejection fraction(LVEF), Killip classification, etc.], and baseline medication (antiplatelet medications, beta-blockers, etc.). The Sacubitril/ Valsartan group was matched 1:1 with the ACEI/ARB group by greedy nearest-neighbor matching (caliper width 0.02), and covariate balance was assessed by standardized mean difference (SMD) (threshold SMD < 0.1). In the matched sample, the corrected hazard ratio (HR) for the 1-year cumulative risk of AF was calculated using a univariate Cox proportional risk regression model (robust variance estimation).

## Results

### Population

**Figure 1:**
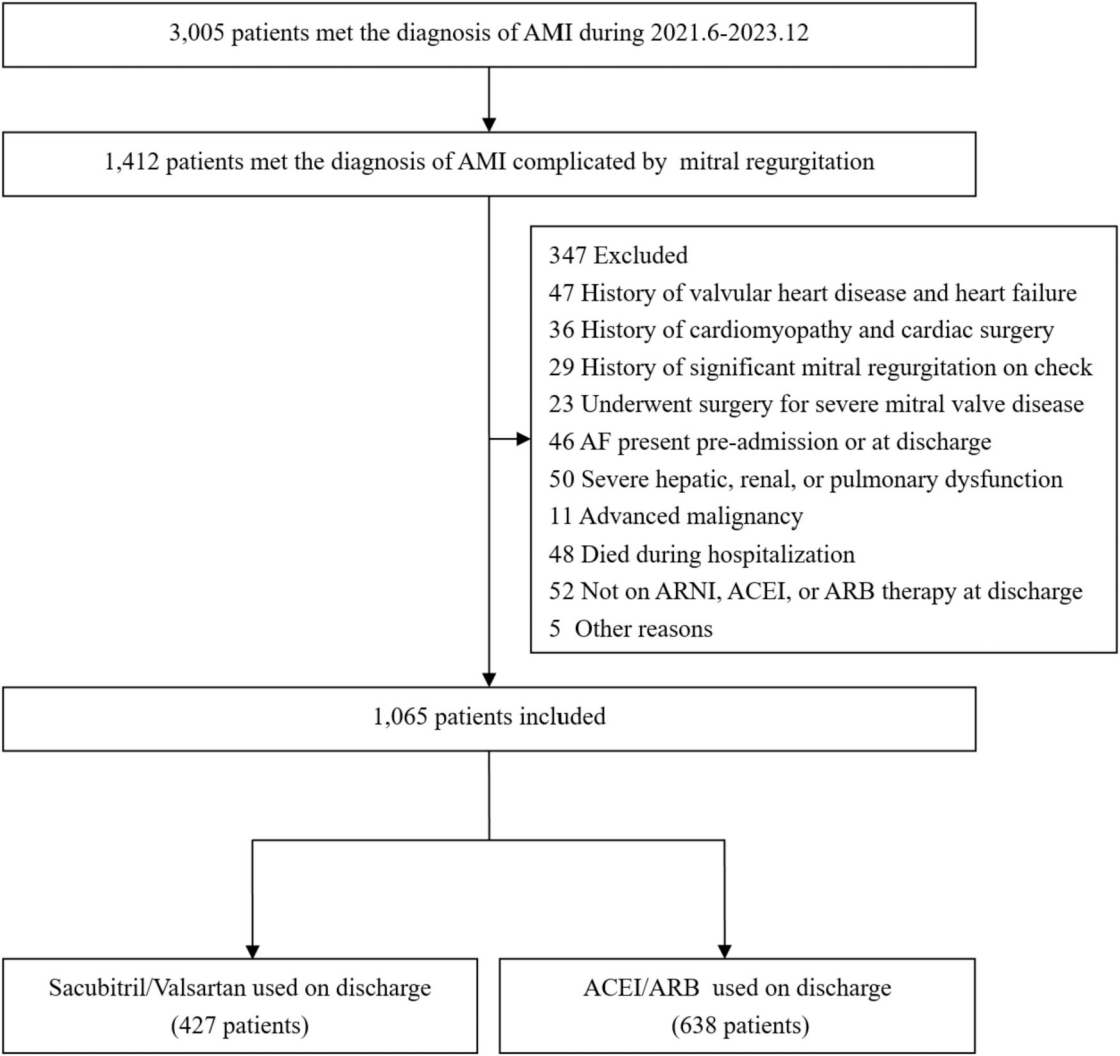
The flow chart of the study. (AMI, acute myocardial infarction; ACEI/ARB, angiotensin-converting enzyme inhibitors/ Angiotensin II Receptor Blockers;ARNI, angiotensin receptor–neprilysin inhibitor).

From June 2021 to December 2023, a total of 3,005 patients with AMI were admitted to the CCU. According to the inclusion and exclusion criteria, the final cohort consisted of 1,065 patients with AMI complicated by MR. The time to Index TTE was 1.6 days (0.9–2.5) in the Sacubitril/Valsartan group and 1.5 days (0.9–2.3) in the ACEI/ARB group, with no significant between-group difference (*P* = 0.732).Among them, 427 patients took sacubitril/valsartan orally, which were referred to as the Sacubitril/Valsartan group, and 638 patients took ACEI/ARB drugs orally, which were referred to as the ACEI/ARB group. The flowchart of patient inclusion and exclusion is shown in Figure 1.

### Baseline characteristics

The baseline characteristics of the two patient groups are summarized in Table 1. Among the 1,065 patients with AMI complicated by MR, significant differences in baseline features were observed between those in the Sacubitril/Valsartan group (*n* = 1. 427) and the ACEI/ARB group (*n* = 638). Patients in the Sacubitril/Valsartan group were older (66.9 ± 11.5 years vs. 65.4 ± 11.8 years, *P* = 0.042) and had higher baseline levels of low-density lipoprotein cholesterol (LDL-C: 3.19 ± 0.55 vs. 3.10 ± 0.57 mmol/L, *P* = 0.010), peak cardiac troponin I [cTnI: 31.5 (24.9–41.8) vs. 24.5 (17.8–39.0) ng/ml, *P* < 0.001], and NT-proBNP [2,240 (1,728–3,310) vs. 1,935 (1,410–2,490) pg/ml, *P* = 0.024] compared with the ACEI/ARB group, suggesting a greater extent of baseline myocardial injury and heart failure burden. Additionally, the Sacubitril/Valsartan group had a lower left ventricular ejection fraction (LVEF: 46.4 ± 4.9% vs. 48.6 ± 5.0%, *P* < 0.001), a higher proportion of moderate-to-severe MR (30.7% vs. 24.5%, *P* = 0.025), and a greater proportion of patients with higher Killip class (II–IV: 74.9% vs. 67.1%, *P* = 0.033). Regarding pharmacotherapy, the use of *β*-blockers was more common in the ACEI/ARB group than in the Sacubitril/Valsartan group (83.1% vs. 75.4%, *P* = 0.002), while the use of other secondary prevention medications, such as aspirin and statins, did not differ significantly between the groups. Notably, during one year of follow-up, the incidence of AF was significantly lower in the Sacubitril/Valsartan group compared to the ACEI/ARB group (10.8% vs. 17.9%, *P* < 0.001), suggesting a potential difference in intervention effect that warrants further investigation.

**Table 1:** Baseline characteristics of the study population.

Variables	Total *n* = 1,065	Sacubitril/Valsartan *n* = 427	ACEI/ARB *n* = 638	*P*
Age, (years)	66.0 ± 11.7	66.9 ± 11.5	65.4 ± 11.8	0.042*
Male, *n* (%)	581 (54.6%)	230 (53.9%)	351 (55.0%)	0.711
BMI, kg/m^2^	24.0 ± 3.6	23.9 ± 3.7	24.1 ± 3.5	0.244
Hypertension, *n* (%)	584 (54.8%)	242 (56.7%)	342 (53.6%)	0.324
Diabetes, *n* (%)	483 (45.1%)	199 (46.6%)	284 (44.5%)	0.502
Smoking, *n* (%)	539 (50.6%)	219 (51.3%)	320 (50.2%)	0.717
History of CAD, *n* (%)	390 (36.6%)	161 (37.7%)	229 (35.9%)	0.548
History of stroke, *n* (%)	267 (25.1%)	105 (24.6%)	162 (25.4%)	0.767
History of CKD, *n* (%)	51 (4.8%)	23 (5.4%)	28 (4.4%)	0.455
History of COPD, *n* (%)	246 (23.1%)	105 (24.6%)	141 (22.1%)	0.345
LDL-C, mmol/L	3.14 ± 0.56	3.19 ± 0.55	3.10 ± 0.57	0.010*
HDL-C, mmol/L	0.96 ± 0.20	0.95 ± 0.18	0.97 ± 0.21	0.086
TG, mmol/L	1.66 ± 0.50	1.65 ± 0.48	1.67 ± 0.51	0.471
TC, mmol/L	5.08 ± 1.08	5.10 ± 1.08	5.07 ± 1.09	0.648
LP(a), mg/L	277 (182,373)	275 (196,374)	278 (175,370)	0.195
UA, μmol/L	349 (283,419)	355 (285,424)	346 (278,417)	0.201
HbA1c, %	6.07 ± 1.19	6.15 ± 1.35	6.01 ± 1.08	0.060
Scr, μmol/L	80.3 ± 18.2	81.6 ± 17.0	79.4 ± 19.0	0.064
Peak cTnI, ng/ml	27.5 (20.0,40.2)	31.5 (24.9,41.8)	24.5 (17.8,39.0)	0.000#
Peak CK, U/L	1,185 (1006,1383)	1,194 (1047,1368)	1,175 (973,1392)	0.150
Peak CK-MB, U/L	105 (95,122)	107 (96,122)	104 (93,121)	0.310
CRP, mg/L	12.2 ± 5.5	12.4 ± 5.5	11.9 ± 5.5	0.110
Hb, g/L	121.0 ± 12.3	120.5 ± 10.7	121.4 ± 13.4	0.244
eGFR, ml/min per1.73 m^2^	98.5 ± 12.5	98.0 ± 12.2	99.0 ± 12.7	0.203
NT-proBNP,pg/ml	2,115 (1524,3250)	2,240 (1728,3310)	1,935 (1410,2490)	0.024*
LVEF, %	47.6 ± 5.0	46.4 ± 4.9	48.6 ± 5.0	0.000^#^
HR, (bpm)(admission)	74.3 ± 14.3	74.9 ± 14.1	74.0 ± 14.5	0.297
SBP, mmHg(admission)	122.4 ± 18.0	123.3 ± 17.7	121.5 ± 18.5	0.112
DBP, mmHg(admission)	75.8 ± 15.6	76.6 ± 15.5	75.0 ± 15.8	0.113
STEMI, *n* (%)	457 (42.9%)	176 (41.2%)	281 (44.0%)	0.361
PCI, *n* (%)	940 (88.3%)	382 (89.5%)	558 (87.5%)	0.320
Killip classification, *n* (%)				0.033*
I	317 (29.8%)	107 (25.1%)	210 (32.9%)	
II	603 (56.6%)	255 (59.7%)	348 (54.5%)	
III	110 (10.3%)	47 (11.0%)	63 (9.9%)	
IV	35 (3.3%)	18 (4.2%)	17 (2.7%)	
MR, *n* (%)				0.025*
Mild	778 (73.1%)	296 (69.3%)	482 (75.5%)	
Moderate and severe	287 (26.9%)	131 (30.7%)	156 (24.5%)	
Medication at discharge, *n* (%)				
Aspirin	979 (91.9%)	384 (89.9%)	595 (93.3%)	0.051
Clopidogrel	910 (85.4%)	361 (84.5%)	549 (86.1%)	0.494
Statins	990 (93.0%)	399 (93.4%)	591 (92.6%)	0.613
*β*-blocker	852 (80.0%)	322 (75.4%)	530 (83.1%)	0.002*
Diuretics	218 (20.5%)	77 (18.0%)	141 (22.1%)	0.107
AF within 1 year, n(%)	160 (15.0%)	46 (10.8%)	114 (17.9%)	0.001*

ACEI, angiotensin-converting enzyme inhibitors; ARB, Angiotensin II Receptor Blockers; BMI, body mass index; CAD, coronary artery disease; CKD, chronic kidney disease; COPD, chronic obstructive pulmonary disease; LDL-C, low-density lipoprotein cholesterol; HDL-C, high-density lipoprotein cholesterol; TG, triglycerides; TC, total cholesterol; LP(a), lipoprotein(a); UA, uric acid; HbA1c, hemoglobin A1c; Scr, serum creatinine; cTnI, cardiac troponin I; CK, creatine kinase; CK-MB, creatine kinase-myocardial band; CRP, C-reactive protein; Hb, hemoglobin; eGFR, estimated glomerular filtration rate; NT-proBNP, N-terminal pro-B-type natriuretic peptide; LVEF, left ventricular ejection fraction; HR, heart rate; BP, blood pressure; AMI, acute myocardial infarction; STEMI, ST-elevation myocardial infarction; PCI, percutaneous coronary intervention; MR, mitral regurgitation; AF, atrial fibrillation.

(*P < 0.05; ^#^*P* < 0.001).

In the overall cohort, the Sacubitril/Valsartan group (*n* = 427) had 46 incident AF events, 5 deaths, and 11 losses to follow-up, with 365 patients administratively censored at 1 year; total person-years were 400.67 and median follow-up was 1 year. The ACEI/ARB group (*n* = 638) had 114 incident AF events, 9 deaths, and 18 losses to follow-up, with 497 patients administratively censored at 1 year; total person-years were 579.97 and median follow-up was 1 year.

### Effect of early use of sacubitril/valsartan on the incidence of AF

In the Sacubitril/Valsartan group (*n* = 427), 46 AF events were identified, of which 32 (69.6%) were detected by 12-lead ECG and 14 (30.4%) by Holter monitoring. In the ACEI/ARB group (*n* = 638), 114 AF events were identified, with 77 (67.5%) detected by 12-lead ECG and 37 (32.5%) by Holter. The distribution of AF detection by modality (12-lead ECG vs. Holter) did not differ significantly between groups (*P* = 0.804).Regarding monitoring intensity, 290 patients (67.9%) in the Sacubitril/Valsartan group underwent at least one Holter within 1 year, totaling 7,464 monitoring hours. In the ACEI/ARB group, 413 patients (64.7%) underwent at least one Holter monitoring, totaling 10,320 h. The proportion of patients completing at least one Holter monitoring did not differ significantly between groups (*P* = 0.283).

All-cause mortality did not differ significantly between groups: 16 deaths (3.7%) in the Sacubitril/Valsartan group vs. 29 deaths (4.5%) in the ACEI/ARB group (*P* = 0.526). Among decedents, 5 in the Sacubitril/Valsartan group and 9 in the ACEI/ARB group had no AF prior to death; all remaining deaths were preceded by AF. In contrast, heart failure rehospitalization occurred less frequently in the Sacubitril/Valsartan group than in the ACEI/ARB group—51 patients (11.9%) vs. 107 patients (16.8%), respectively—with a statistically significant between-group difference (*P* = 0.030).

**Figure 2:**
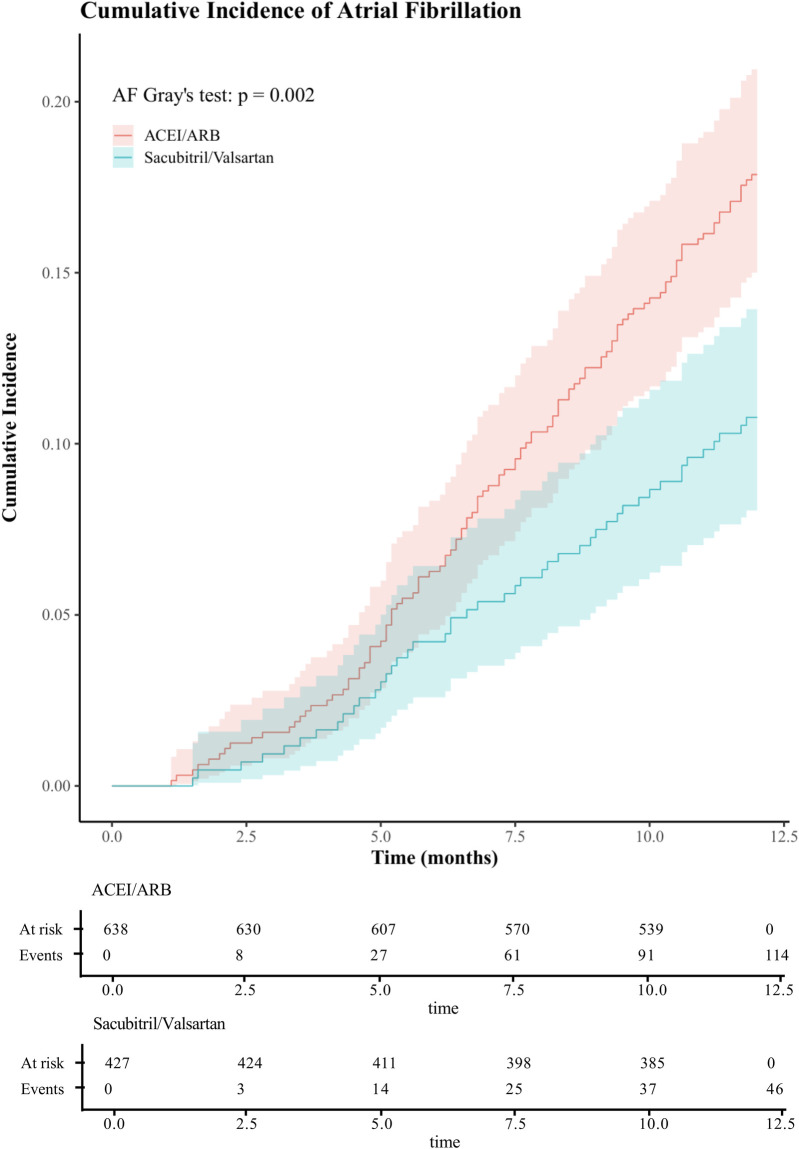
One-year cumulative incidence of atrial fibrillation in acute myocardial infarction with mitral regurgitation: sacubitril/valsartan vs ACEI/ARB, assessed by CIF and Gray's test. (ACEI/ARB, angiotensin-converting enzyme inhibitors/ angiotensin II receptor blockers;CIF, cumulative incidence function).

**Figure 3:**
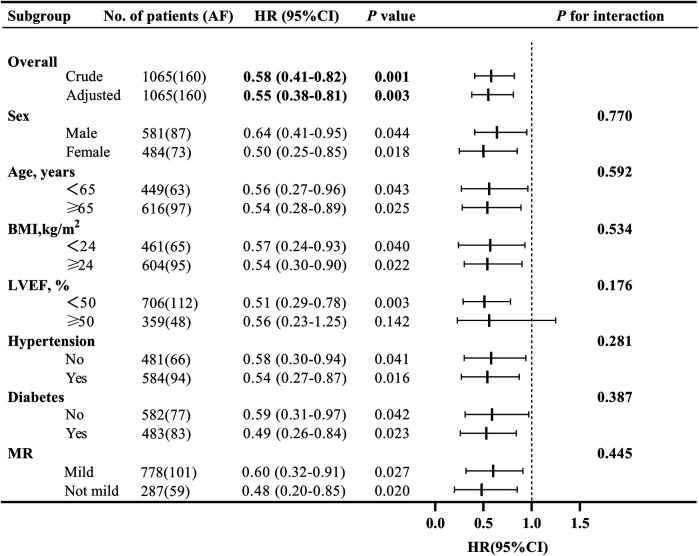
Forest plot of subgroup analyses for the effect of early sacubitril/valsartan use on the incidence of atrial fibrillation at 1-year in acute myocardial infarction with mitral regurgitation. (BMI, body mass index; MR, mitral regurgitation; LVEF, left ventricular ejection fraction; HR, hazard ratio; CI, confidence interval).

Treating death as a competing event, the Sacubitril/Valsartan group showed a lower cumulative incidence of new-onset AF than the ACEI/ARB group over a median follow-up of 12 months. In Fine–Gray subdistribution hazard models adjusting for confounders, sacubitril/valsartan therapy was associated with a reduced cumulative incidence of AF [subdistribution hazard ratios (sHR) = 0.56, 95% CI 0.39–0.81; *P* = 0.002]. As shown in Figure 2 and Supplementary Figure S1, the 1-year cumulative incidence functions (CIFs) were 10.8% for sacubitril/valsartan and 17.9% for ACEI/ARB (Gray's test *P* = 0.002). Given death as a competing event, we fitted Cox models for the outcome of interest and verified the proportional hazards assumption using Schoenfeld residuals; the global test showed no violation (global *χ*2 = 46.74,*P* = 0.215).In both univariate and multivariate Cox proportional hazards analyses across five models (Table 2), the use of sacubitril/valsartan remained consistently associated with a reduced risk of AF, with hazard ratios (HRs) ranging from 0.55 to 0.60 (*P* < 0.05 for all). After adjusting for all covariates listed in Table 1, early use of sacubitril/valsartan was associated with a 45% reduction in the risk of AF compared with the ACEI/ARB group [HR = 0.55; 95% confidence interval (CI): 0.38–0.81; *P* = 0.003]. Subgroup analyses assessed by forest plots further demonstrated the consistency of the treatment effect across prespecified patient subgroups, including age, sex, severity of MR, LVEF, Killip class, and diabetes status (Figure 3). The HRs for AF risk in all subgroups favored sacubitril/valsartan, and no significant interactions were detected between treatment effect and any of the subgroup variables (all interaction *P* values > 0.05).

**Table 2:** Univariate and multivariable Cox proportional risk analysis of sacubitril/valsartan against the incidence of atrial fibrillation within 1 year.

Variable	Group	Hazard Ratio	95% CI	*P*-value
Unadjusted	ACEI/ARB	Reference		
	Sacubitril/Valsartan	0.58	(0.41–0.82)	0.001*
Model 1	ACEI/ARB	Reference		
	Sacubitril/Valsartan	0.57	(0.41–0.81)	0.003*
Model 2	ACEI/ARB	Reference		
	Sacubitril/Valsartan	0.60	(0.42–0.84)	0.001*
Model 3	ACEI/ARB	Reference		
	Sacubitril/Valsartan	0.55	(0.38–0.78)	0.001*
Model 4	ACEI/ARB	Reference		
	Sacubitril/Valsartan	0.57	(0.39–0.82)	0.002*
Model 5	ACEI/ARB	Reference		
	Sacubitril/Valsartan	0.55	(0.38–0.81)	0.003*

Model 1: Adjusted for age, sex.

Model 2: Adjusted for model 1 +(BMI, hypertension, diabetes, smoking, history of CAD, history of stroke, history of CKD, history of COPD).

Model 3: Adjusted for model 2 + (LDL-C, HDL-C, TG, TC, LP-a, UA, HbA1c, Scr, peak cTnI, peak CK, peak CK-MB, CRP, Hb, eGFR, NT-proBNP).

Model 4: Adjusted for model 3 +(LVEF, HR, SBP, DBP, type of AMI, PCI).

Model 5: Adjusted for model 4 +(Killip classification, MR, medication at discharge).

(*P < 0.05).

### Sensitivity analyses using propensity score matching

**Figure 4:**
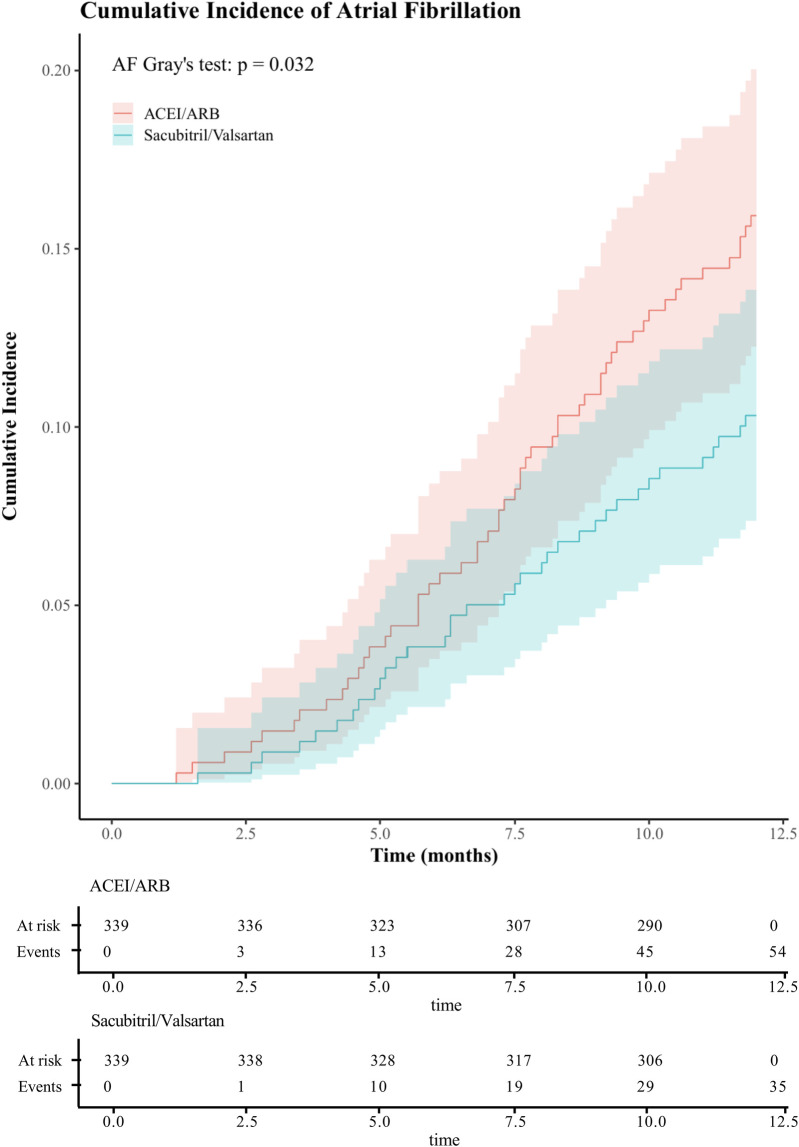
One-year cumulative incidence of atrial fibrillation after propensity score matching in acute myocardial infarction with mitral regurgitation: sacubitril/valsartan vs ACEI/ARB, assessed by CIF and Gray's test. (ACEI/ARB, angiotensin-converting enzyme inhibitors/ angiotensin II receptor blockers;CIF, cumulative incidence function).

After PSM yielded 339 matched pairs, the one-year CIFs for new-onset AF and for death showed consistent separation between treatment groups (Figure 4 and Supplementary Figure S2), with sacubitril/valsartan exhibiting a lower incidence of AF than ACEI/ARB (Gray's test *P* = 0.032), while the curves for death overlapped (Gray's test *P* = 0.995). The two groups had comparable baseline characteristics after matching (Supplementary Table S1), and balance diagnostics were shown in Supplementary Figure S3 (all SMDs <0.1). In the univariable Cox model, sacubitril/valsartan was associated with a reduced hazard of AF (HR = 0.63, 95% CI 0.41–0.96; *P* = 0.030). The proportional hazards assumption was evaluated using Schoenfeld residuals; the global test indicated no violation (global *χ*2 = 53.55, *P* = 0.074). This association remained significant when accounting for the competing risk of death using the Fine–Gray method (sHR = 0.63, 95% CI 0.40–0.94; *P* = 0.025). These findings, concordant across specifications, reinforce the robustness of the association between early sacubitril/valsartan use and reduced one-year AF.

Among patients who underwent at least one Holter monitoring within 1 year, a sensitivity analysis was performed using a Cox proportional hazards model adjusted for covariates. Compared with the ACEI/ARB group, the Sacubitril/Valsartan group exhibited a significantly lower risk of AF (HR = 0.53, 95% CI 0.29–0.97; *P* = 0.039). This finding is consistent with the primary analysis, supporting the robustness of the overall results.

### Exploratory biomarker analysis: baseline NT-proBNP

**Figure 5:**
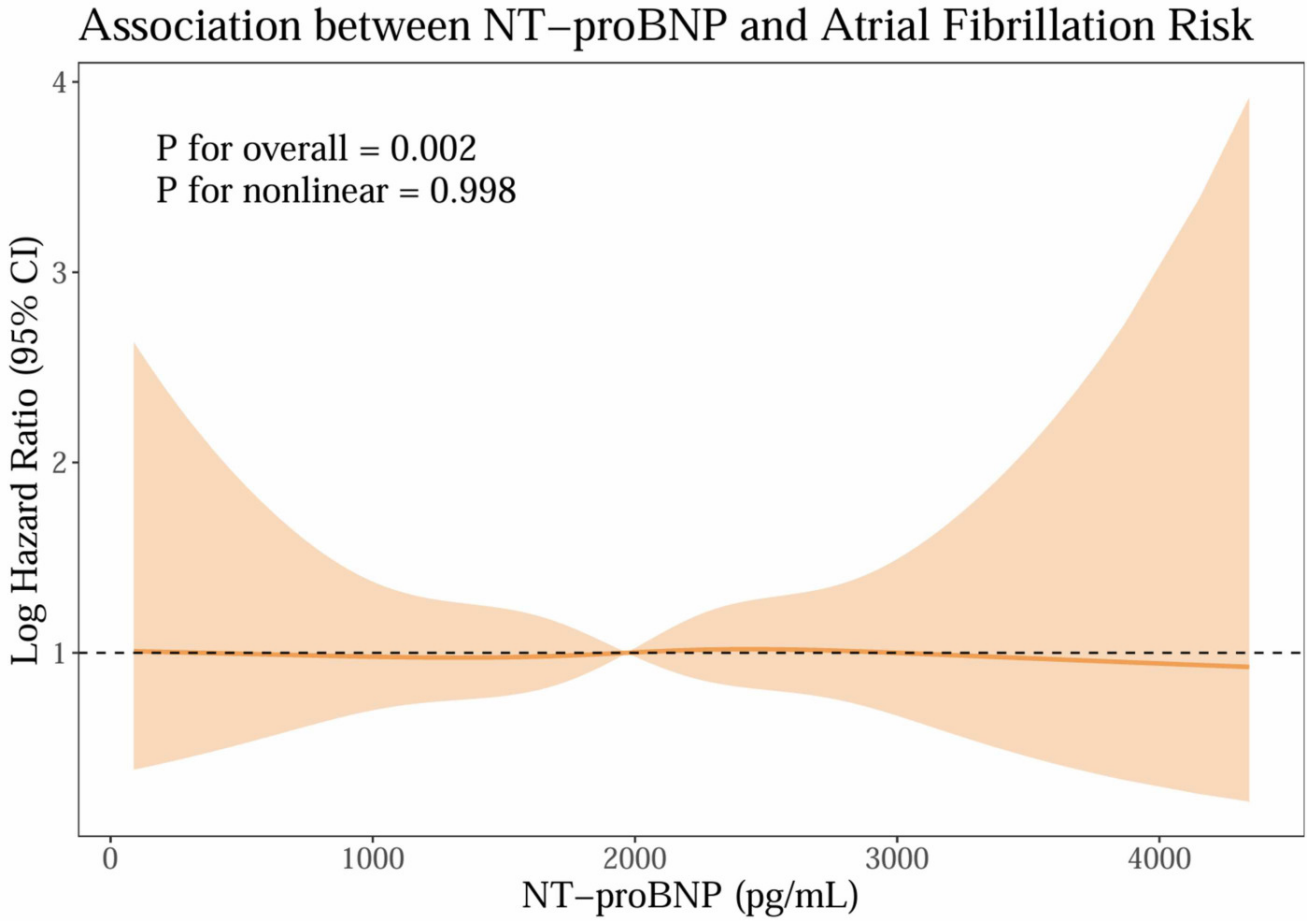
Exploratory biomarker analysis: baseline NT-proBNP. (NT-proBNP; n-terminal pro-b-type natriuretic peptide).

To explore the dose–response association between baseline NT-proBNP and new-onset AF, we fitted restricted cubic spline Cox models within the matched cohort. As shown in Figure 5, higher NT-proBNP levels were associated with a greater risk of AF, with a significant overall association (*P*_overall_=0.002) and no evidence of nonlinearity (*P*_nonlinear_=0.998), suggesting an approximately linear increase in risk. This biomarker–outcome relationship provides biological plausibility for the observed association between early sacubitril/valsartan use and lower AF risk, given its pharmacological effect in lowering NT-proBNP levels. Proportional hazards were satisfied (global *χ*2 = 53.55, *P* = 0.074).

### Safety outcomes

In the safety analysis, adverse events were systematically assessed throughout follow-up. The incidence of hypotension was higher in the Sacubitril/Valsartan group than in the ACEI/ARB group (31 [7.3%] vs. 27 [4.2%]; *P* = 0.033). In contrast, there were no statistically significant between-group differences in worsening renal function (15 [3.5%] vs. 29 [4.5%]; *P* = 0.407) or hyperkalemia (14 [3.3%] vs. 30 [4.7%]; *P* = 0.253). No angioedema events occurred in either group. Regarding time to first event, the earliest hypotension occurred 15 days post-discharge in the Sacubitril/Valsartan group, whereas the first events of worsening renal function and hyperkalemia were observed 6 and 30 days post-discharge, respectively, in the ACEI/ARB group. Collectively, these findings indicate a higher risk of hypotension with sacubitril/valsartan, while worsening renal function, hyperkalemia and angioedema events were comparable between treatment strategies.

## Discussion

In this retrospective cohort study, we compared the one-year incidence of AF between sacubitril/valsartan and ACEI/ARB therapy in patients with AMI complicated by MR. Our findings demonstrate that, compared with ACEI/ARB therapy, early initiation of sacubitril/valsartan was associated with a significantly lower cumulative incidence of AF at one year in this high-risk population. Specifically, sacubitril/valsartan reduced the risk of developing AF by 45% (HR = 0.55; 95% CI: 0.38–0.81; *P* = 0.003), underscoring its potential benefit over conventional renin–angiotensin system inhibitors in post-AMI patients with concomitant MR.

Accumulating evidence from multiple clinical studies suggests that sacubitril/valsartan may reduce the incidence of AF. For instance, a real-world observational study (23) in adult cardiac surgery patients found that sacubitril/valsartan was associated with a significantly lower incidence of postoperative AF and more pronounced LVEF recovery compared with usual care. Similarly, Chen et al. demonstrated (24) that sacubitril/valsartan significantly reduced the recurrence rate of AF after electrical cardioversion in patients with persistent AF. Notably, the novelty of our study lies in its focus on a high-risk population—patients with AMI complicated by MR. Our findings extend the preventive value of sacubitril/valsartan on AF to this population, who are at particularly high risk for arrhythmia. This benefit may be attributable to the dual mechanism of sacubitril/valsartan, which combines renin–angiotensin system inhibition with enhancement of the natriuretic peptide system (25), thereby providing more comprehensive modulation of cardiac electrophysiological stability.

The beneficial effect of sacubitril/valsartan on ventricular remodeling after AMI has been well established. After 1:1 PSM, 339 patients were included in the Sacubitril/Valsartan group and 339 in the ACEI/ARB group, with comparable baseline characteristics. At baseline, the proportion of moderate and severe MR was similar between groups: 28.0% in the Sacubitril/Valsartan group vs. 28.3% in the ACEI/ARB group (*P* = 0.932). At 6 months, moderate and severe MR was observed in 16.1% of the Sacubitril/Valsartan group vs. 22.6% of the ACEI/ARB group (*P* = 0.032). These findings suggest that, compared with ACEI/ARB, ARNI may reduce functional MR burden, potentially via favorable effects on ventricular remodeling. MR after AMI is broadly classified into two mechanisms: ischemic/functional and structural. The ischemic/functional form stems from left ventricular remodeling that induces leaflet tethering and inadequate coaptation, with the valve apparatus typically intact; in contrast, structural MR results from direct damage to the apparatus, such as chordal or papillary muscle rupture or leaflet perforation. These mechanisms differ substantially in therapeutic strategy and prognosis. Notably, in the setting of AMI, ischemic/functional MR predominates.In a randomized controlled trial involving patients with AMI complicated by moderate-to-severe MR, sacubitril/valsartan demonstrated significantly greater improvements in LVEF and ventricular volume parameters compared with ACEI therapy (15). This finding is consistent with the meta-analysis by Gao et al. (26), which showed that sacubitril/valsartan significantly reduced left ventricular end-systolic volume and left ventricular end-diastolic volume in patients with post-AMI heart failure, thereby reversing ventricular dilation. These clinical observations are further corroborated by experimental studies. For example, Li et al. reported in a rabbit model of AF that sacubitril/valsartan attenuated atrial and right ventricular enlargement and mitigated myocardial fibrosis by reducing collagen deposition (27). Mechanistically, these effects may be attributed to the suppression of angiotensin II-induced activation of p-Smad2/3, p-JNK, and p-p38MAPK signaling pathways, thereby blocking the activation of pro-fibrotic factors (28). The present study extends these findings by demonstrating a significant reduction in the incidence of AF with sacubitril/valsartan, possibly mediated by improvements in ventricular remodeling that reduce mechanical stretch and electrical heterogeneity, thus indirectly attenuating the risk of AF.

Beyond its established effects on ventricular remodeling, sacubitril/valsartan appears to exert crucial antiarrhythmic benefits through atrial reverse remodeling. Suo et al. (29) demonstrated via echocardiographic assessment that sacubitril/valsartan enhanced the systolic function of the left atrium and left atrial appendage, thereby reducing blood stasis and potentially lowering the risks of thrombus formation and AF recurrence. Furthermore, studies in post–catheter ablation patients revealed that sacubitril/valsartan reduced the extent of left atrial fibrosis, as assessed by cardiac MRI, and improved electrical conduction heterogeneity, which may contribute to more durable maintenance of sinus rhythm (30, 31). While our study did not directly assess atrial fibrosis, the cumulative literature supports the hypothesis that sacubitril/valsartan may suppress atrial structural remodeling by targeting multiple fibrotic pathways, including inhibition of TGF-*β*/Smad signaling, thereby reducing the substrate required for both the initiation and perpetuation of AF.

There is substantial mechanistic evidence from basic science studies supporting the clinical benefits observed in this study. Li et al. demonstrated *in vitro* that sacubitril/valsartan inhibits angiotensin II–induced atrial fibroblast activation and reduces collagen I/III synthesis (28), an effect mediated by dual blockade of the p-Smad2/3, p-JNK, and p-p38MAPK signaling pathways. In animal models, sacubitril/valsartan has been shown to reverse AF–induced electrical remodeling (27)—such as shortening of the action potential duration—as well as structural remodeling characterized by interstitial fibrosis. Furthermore, Wei et al. reported that sacubitril/valsartan activates the PPARs pathway (32), leading to upregulation of FGF21 signaling and attenuation of myocardial ischemia–reperfusion injury, which may indirectly improve the atrial microenvironment following AMI. Collectively, these basic research findings not only elucidate the molecular mechanisms underlying the reduction in AF incidence observed in our cohort but also underscore the potential for sacubitril/valsartan as a novel therapeutic strategy in arrhythmia prevention and management, guiding future translational and clinical investigations.

In summary, the reduction in AF incidence observed with sacubitril/valsartan appears to result from multiple synergistic mechanisms. First, by inhibiting the renin–angiotensin system, sacubitril/valsartan attenuates the pro-fibrotic effects of angiotensin II while simultaneously enhancing the natriuretic peptide system's anti-fibrotic and natriuretic actions, thereby alleviating hemodynamic stress (33). Second, it directly suppresses myocardial fibrosis and electrical remodeling through modulation of key signaling pathways, including p-Smad2/3, p-JNK, and p-p38MAPK (32). Third, emerging evidence suggests that activation of the PPARs/FGF21 axis by sacubitril/valsartan mitigates oxidative stress and inflammation, providing further cardiomyocyte protection (26, 34, 35). Future multicenter randomized controlled trials are warranted to determine the optimal timing and dosing of early intervention, as well as to explore the therapeutic potential of sacubitril/valsartan in other arrhythmia subtypes, such as ventricular tachycardia.

In exploratory analyses, baseline NT-proBNP showed a positive, approximately linear association with incident AF (Figure 5), with a significant overall effect and no evidence of nonlinearity. This dose–response pattern is directionally consistent with our primary finding that early sacubitril/valsartan use was associated with a lower risk of AF compared with ACEI/ARB, given the established ability of sacubitril/valsartan to reduce NT-proBNP levels. While these observations cannot establish causality, the biomarker–outcome relationship provides biological plausibility and supports the coherence of the main result. The finding also aligns with prior evidence linking higher natriuretic peptide levels to atrial stretch, structural remodeling, and heightened AF susceptibility, thereby situating our results within a well-described pathophysiological framework.

Moreover, the restricted cubic splines (RCS) association persisted across clinically relevant ranges of NT-proBNP and appeared robust in the matched cohort adjusted for prespecified covariates. Taken together, these data suggest that patients with higher neurohormonal activation, as indexed by NT-proBNP, experience greater AF risk, and that therapies known to lower NT-proBNP may be directionally aligned with AF risk reduction. Nonetheless, residual confounding, reverse causation, and indication bias remain possible in an observational design, and the present analyses should be interpreted as associative rather than causal.

Future work should test whether NT-proBNP dynamics mediate part of the observed association using longitudinal biomarker measurements and formal mediation analysis, and whether treatment effects or associations vary by baseline NT-proBNP strata in adequately powered randomized or quasi-experimental settings.

### Limitations

This study has several important limitations. First, its retrospective design inherently introduces selection bias and potential information bias, despite the application of PSM to minimize confounding. Second, the single-center nature of the study and the relatively limited sample size may restrict the generalizability of our findings to broader populations. Third, although multiple baseline characteristics were adjusted, residual confounding from unmeasured or unknown variables cannot be fully excluded. Fourth, asymptomatic subclinical AF not detected by the scheduled follow-up surveillance could have been missed, potentially leading to an underestimation of the true incidence of AF. Additionally, while our findings are supported by existing preclinical and clinical evidence, prospective multicenter randomized trials are needed to validate these results. These limitations should be considered when interpreting the implications of our research.

## Conclusion

In patients with AMI complicated by MR, early sacubitril/valsartan use, compared with ACEI/ARB therapy, was associated with a lower 1-year risk of new-onset AF. These observational findings suggest a potential clinical benefit in this high-risk population, which warrants confirmation in prospective randomized studies.

## Data Availability

The original contributions presented in the study are included in the article/Supplementary Material, further inquiries can be directed to the corresponding author.

## Supplementary material

Supplementary Figure S1 One-year cumulative incidence of atrial fibrillation and death in acute myocardial infarction with mitral regurgitation: Sacubitril/Valsartan vs ACEI/ARB, assessed by CIF and Gray's test.(ACEI/ARB, angiotensin-converting enzyme inhibitors/ angiotensin II receptor blockers;CIF, cumulative incidence function).

Supplementary Figure S2 One-year cumulative incidence of atrial fibrillation and death after propensity score matching in acute myocardial infarction with mitral regurgitation: Sacubitril/Valsartan vs ACEI/ARB, assessed by CIF and Gray's test.(ACEI/ARB, angiotensin-converting enzyme inhibitors/angiotensin II receptor blockers;CIF, cumulative incidence function).

Supplementary Figure S3 MD, standardized mean difference; IPTW, inverse probability of treatment weighting; SMRW, standardized mortality ratio weighting; PA, propensity adjustment;Ow, overlap weighting).
