# Supplementary material for: Early sacubitril/valsartan use associated with reduced atrial fibrillation risk in patients with acute myocardial infarction complicated by mitral regurgitation: a retrospective cohort study
Source: Front Cardiovasc Med. 2025 Sep 26;12:1658746. doi: 10.3389/fcvm.2025.1658746 (PMC12510935; doi:10.3389/fcvm.2025.1658746)
Supplement: Supplementary file 1 [file Table1.pdf]

**Supplementary Table 1** Baseline characteristics of the study population after propensity score matching.

| Variables | Sacubitril/Valsartan n=339 | ACEI/ARB n=339 | *P* value | SMD |
|---|---|---|---|---|
| Age,(years) | 66.4±11.8 | 66.1±11.6 | 0.721 | 0.024 |
| Male,n(%) | 186(54.9%) | 197(58.1%) | 0.394 | 0.029 |
| BMI,kg/m$^2$ | 24.0±3.7 | 24.2±3.4 | 0.613 | 0.004 |
| Hypertension,n(%) | 192(56.6%) | 188(55.5%) | 0.757 | 0.023 |
| Diabetes,n(%) | 154(45.4%) | 158(46.6%) | 0.758 | 0.082 |
| Smoking,n(%) | 170(50.1%) | 167(49.3%) | 0.818 | 0.006 |
| History of CAD,n(%) | 126(37.2%) | 121(35.7%) | 0.690 | 0.024 |
| History of stroke,n(%) | 84(24.8%) | 88(26.0%) | 0.724 | 0.067 |
| History of CKD,n(%) | 14(4.1%) | 16(4.7%) | 0.709 | 0.014 |
| History of COPD,n(%) | 81(23.9%) | 83(24.5%) | 0.858 | 0.048 |
| LDL-C,mmol/L | 3.17±0.55 | 3.20±0.56 | 0.482 | 0.040 |
| HDL-C,mmol/L | 0.96±0.18 | 0.96±0.21 | 0.862 | 0.041 |
| TG,mmol/L | 1.66±0.49 | 1.65±0.51 | 0.684 | 0.019 |
| TC, mmol/L | 5.09±1.07 | 5.11±1.09 | 0.789 | 0.038 |
| LP(a),mg/L | 276(192,363) | 277(178,380) | 0.498 | 0.047 |
| UA, µmol/L | 352(284,424) | 350(292,429) | 0.514 | 0.010 |
| HbA1c,% | 6.14±1.37 | 6.09±1.06 | 0.623 | 0.004 |
| Scr, µmol/L | 80.7±16.9 | 81.1±18.9 | 0.772 | 0.033 |
| Peak cTnI,ng/ml | 30.0(23.0,39.6) | 30.5(21.5,41.0) | 0.920 | 0.025 |
| Peak CK,U/L | 1185(1044,1365) | 1177(965,1393) | 0.576 | 0.054 |
| Peak CK-MB,U/L | 103(98,119) | 104(96,121) | 0.796 | 0.045 |
| CRP,mg/L | 12.2±5.4 | 12.0±5.4 | 0.590 | 0.027 |
| Hb,g/L | 120.7±10.6 | 120.0± 13.7 | 0.463 | 0.017 |
| eGFR,mL/min per1.73 m$^2$ | 98.3±11.9 | 98.7±12.2 | 0.671 | 0.014 |
| NT-proBNP,pg/ml | 2068(1557,2548) | 2010(1545,2530) | 0.746 | 0.021 |
| LVEF,% | 47.0±4.8 | 47.4±4.9 | 0.320 | 0.028 |
| HR,(bpm)(admission) | 74.4±14.1 | 74.7±14.5 | 0.741 | 0.018 |
| SBP,mmHg(admission) | 122.7±17.5 | 121.4±17.8 | 0.345 | 0.004 |
| DBP,mmHg(admission) | 76.1±15.9 | 75.5±16.2 | 0.625 | 0.057 |
| STEMI,n(%) | 140(41.3%) | 141(41.6%) | 0.938 | 0.047 |
| PCI,n(%) | 300(88.5%) | 299(88.2%) | 0.905 | 0.018 |
| Killip classification,n(%) | | | 0.916 | 0.055 |
| I | 88(26.0%) | 96(28.3%) | | |
| II | 204(60.2%) | 199(58.7%) | | |
| III | 35(10.3%) | 33(9.7%) | | |
| IV | 12(3.5%) | 11(3.2%) | | |
| MR,n(%) | | | 0.932 | 0.020 |
| mild | 244(72.0%) | 243(71.7%) | | |
| moderate and severe | 95(28.0%) | 96(28.3%) | | |
| Medication at discharge,n(%) | | | | |
| Aspirin | 310(91.4%) | 307(90.6%) | 0.687 | 0.022 |
| Clopidogrel | 288(85.0%) | 287(84.7%) | 0.915 | 0.003 |
| Statins | 314(92.6%) | 319(94.1%) | 0.440 | 0.011 |
| β-blocker | 273(80.5%) | 266(78.5%) | 0.505 | 0.015 |
| Diuretics | 56(16.5%) | 55(16.2%) | 0.917 | 0.023 |

ACEI, angiotensin-converting enzyme inhibitors; ARB, Angiotensin II Receptor Blockers; BMI, body mass index; CAD, coronary artery disease; CKD, chronic kidney disease; COPD, chronic obstructive pulmonary disease; LDL-C, low-density lipoprotein cholesterol; HDL-C, high-density lipoprotein cholesterol; TG, triglycerides; TC, total cholesterol; LP(a), lipoprotein(a); UA, uric acid; HbA1c, hemoglobin A1c; Scr, serum creatinine; cTnI, cardiac troponin I; CK, creatine kinase; CK-MB, creatine kinase-myocardial band; CRP, C-reactive protein; Hb, hemoglobin; eGFR, estimated glomerular filtration rate; NT-proBNP, N-terminal pro-B-type natriuretic peptide; LVEF, left ventricular ejection fraction; HR, heart rate; BP, blood pressure; AMI, acute myocardial infarction; STEMI, ST-elevation myocardial infarction; PCI, percutaneous coronary intervention; MR, mitral regurgitation.
