# Supplementary figures and images for: Early sacubitril/valsartan use associated with reduced atrial fibrillation risk in patients with acute myocardial infarction complicated by mitral regurgitation: a retrospective cohort study
Source: Front Cardiovasc Med. 2025 Sep 26;12:1658746. doi: 10.3389/fcvm.2025.1658746 (PMC12510935; doi:10.3389/fcvm.2025.1658746)

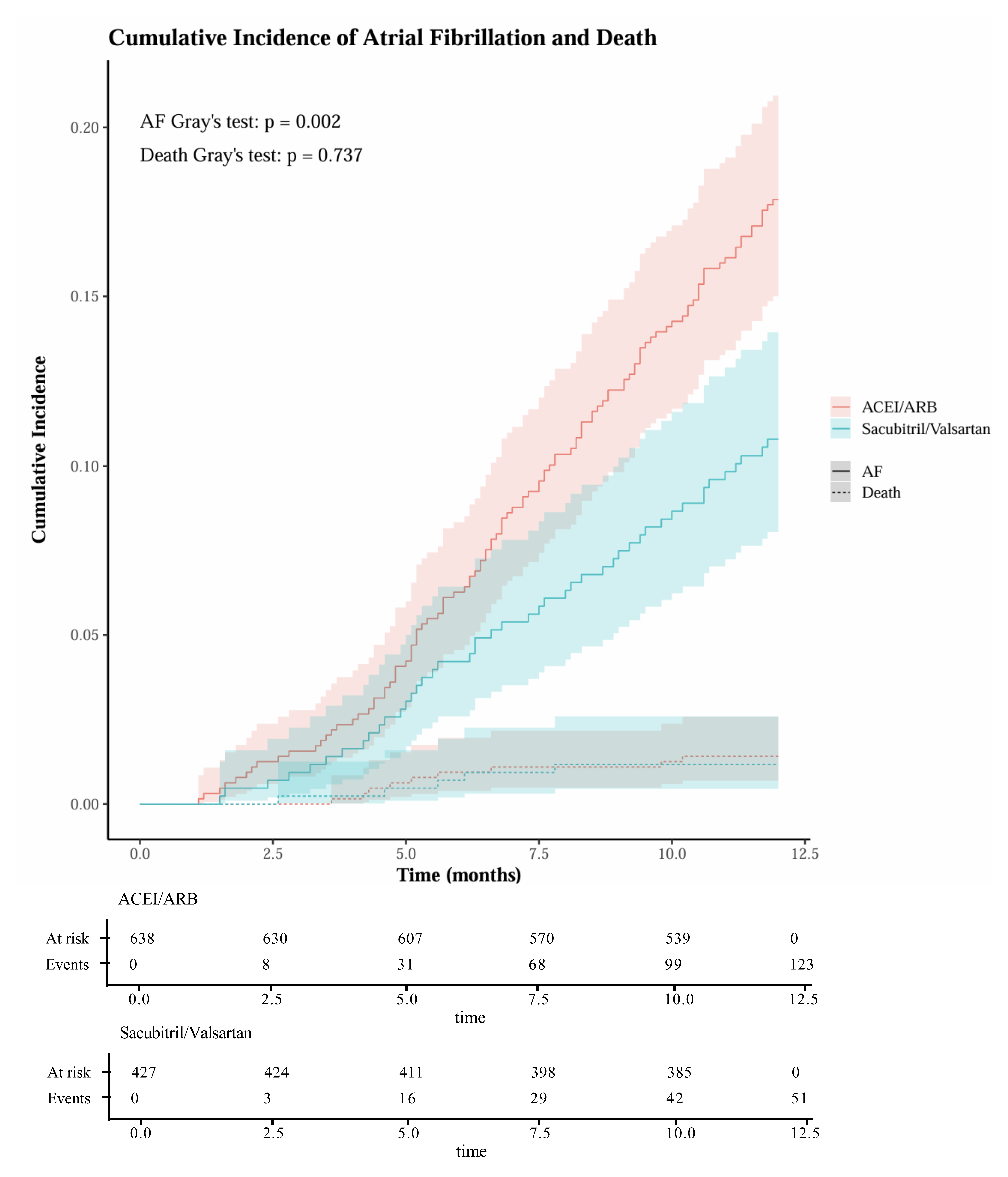

Supplement: Supplementary Figure S1 — One-year cumulative incidence of atrial fibrillation and death in acute myocardial infarction with mitral regurgitation: Sacubitril/Valsartan vs ACEI/ARB, assessed by CIF and Gray's test.(ACEI/ARB, angiotensin-converting enzyme inhibitors/ angiotensin II receptor blockers;CIF, cumulative incidence function). [file Image1.tif]

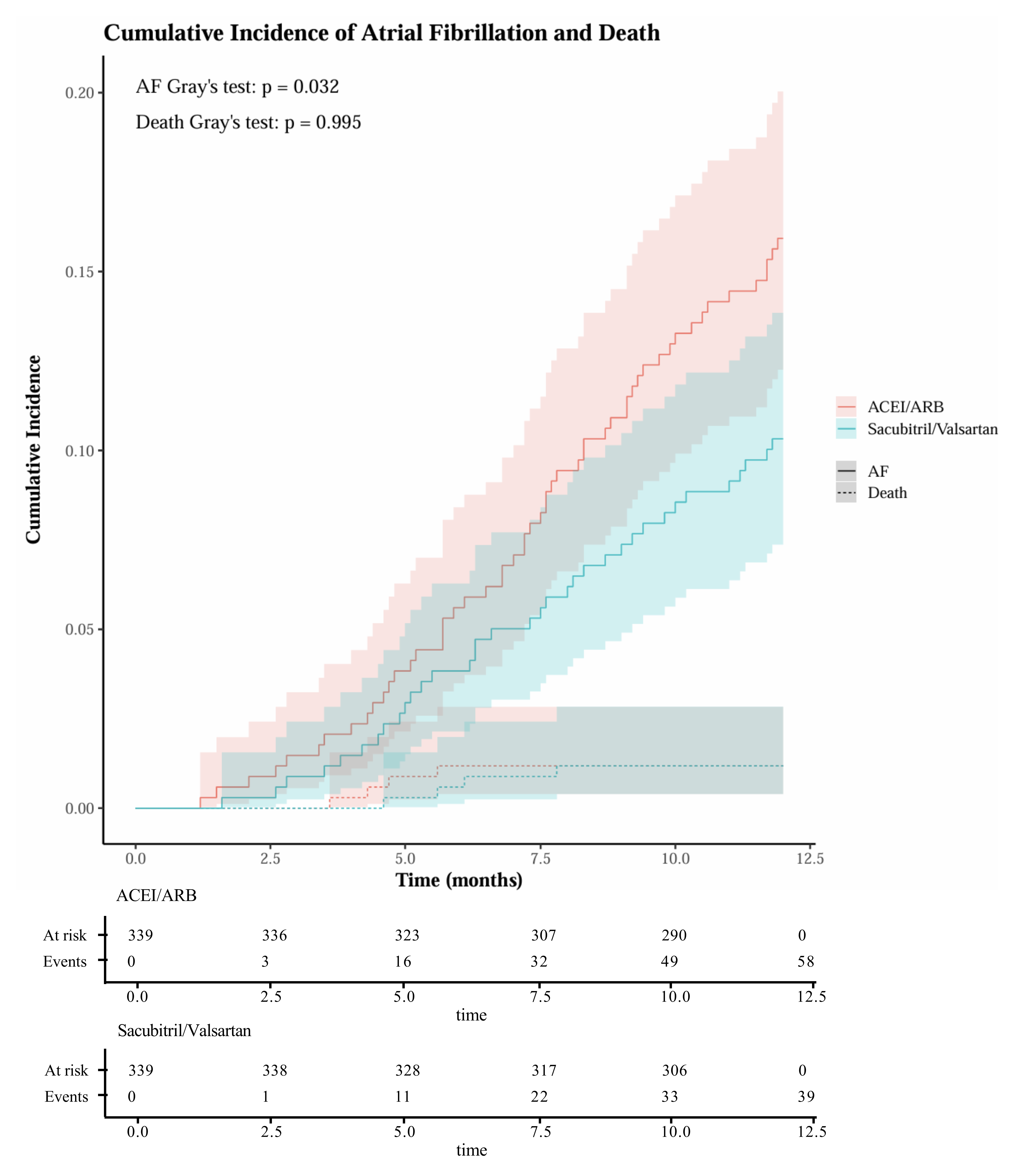

Supplement: Supplementary Figure S2 — One-year cumulative incidence of atrial fibrillation and death after propensity score matching in acute myocardial infarction with mitral regurgitation: Sacubitril/Valsartan vs ACEI/ARB, assessed by CIF and Gray's test.(ACEI/ARB, angiotensin-converting enzyme inhibitors/angiotensin II receptor blockers;CIF, cumulative incidence function). [file Image2.tif]

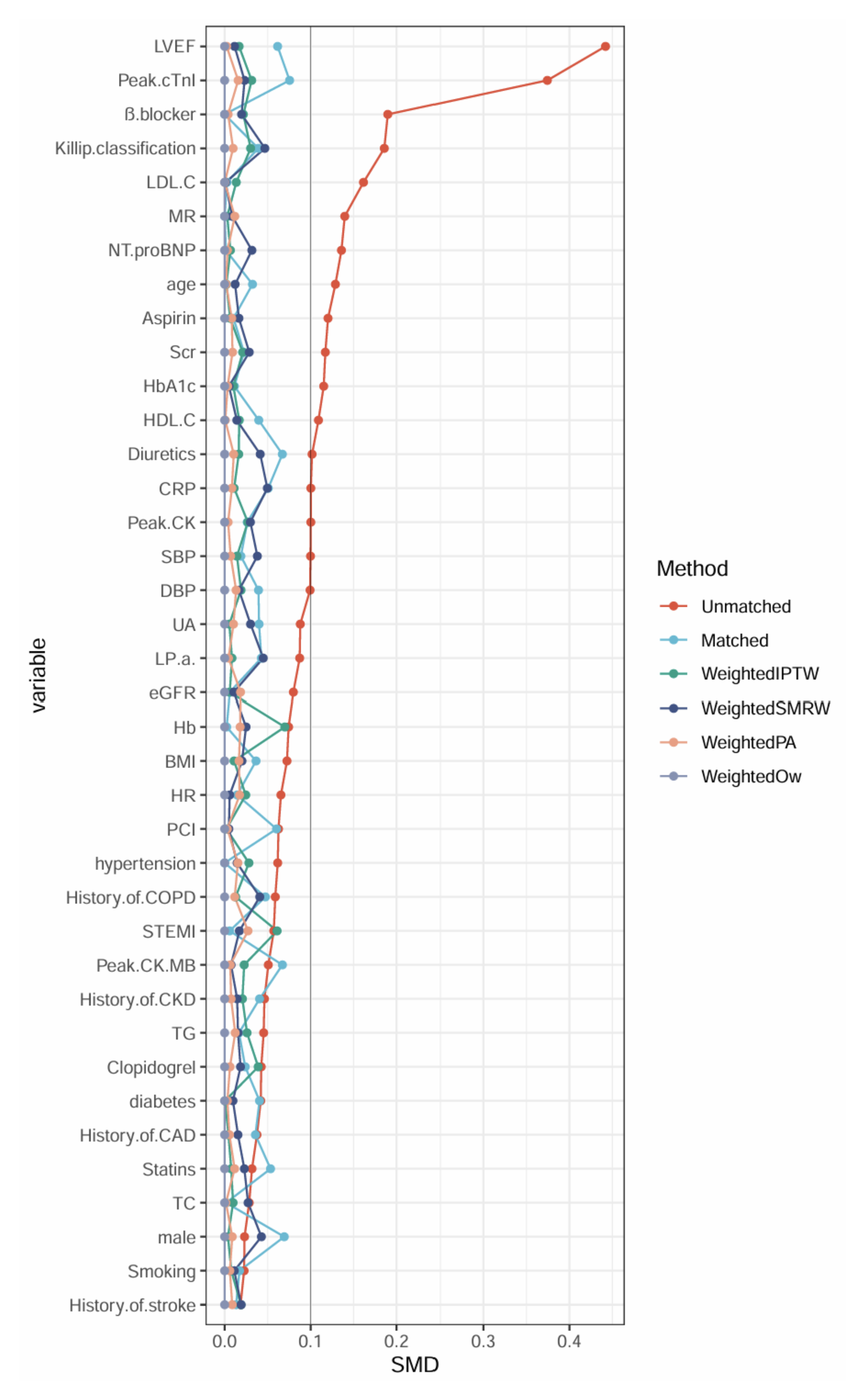

Supplement: Supplementary Figure S3 — MD, standardized mean difference; IPTW, inverse probability of treatment weighting; SMRW, standardized mortality ratio weighting; PA, propensity adjustment;Ow, overlap weighting). [file Image3.tif]
